# Kupffer Cells and Hepatocytes: A Key Relation in the Context of Canine Leishmaniasis

**DOI:** 10.3390/microorganisms12091887

**Published:** 2024-09-13

**Authors:** Armanda Rodrigues, Graça Alexandre-Pires, Ana Valério-Bolas, Telmo Nunes, Isabel Pereira da Fonseca, Gabriela Santos-Gomes

**Affiliations:** 1 Global Health and Tropical Medicine (GHTM), Associate Laboratory in Translation and Innovation towards Global Health, LA-REAL, Instituto de Higiene e Medicina Tropical (IHMT), Universidade NOVA de Lisboa (UNL), 1349-008 Lisbon, Portugal; armanda.rodrigues@ihmt.unl.pt (A.R.); ana.bolas@ihmt.unl.pt (A.V.-B.); 2 CIISA—Centro de Investigação Interdisciplinar em Sanidade Animal, Faculdade de Medicina Veterinária, Universidade de Lisboa, Avenida da Universidade Técnica, 1300-477 Lisbon, Portugal; gpires@fmv.ulisboa.pt (G.A.-P.); ifonseca@fmv.ulisboa.pt (I.P.d.F.); 3 Associate Laboratory for Animal and Veterinary Sciences (AL4AnimalS), 1200-771 Lisbon, Portugal; 4 Microscopy Center, Faculty of Sciences, Universidade de Lisboa, 1749-016 Lisbon, Portugal; telmonunes@hotmail.com

**Keywords:** co-culture, hepatocytes, Kupffer cells, liver immunology, *Leishmania infantum*, canine leishmaniasis

## Abstract

Human zoonotic visceral leishmaniasis (ZVL) and canine leishmaniasis (CanL) constitute a major public and veterinary health concern and are both caused by the infection with the protozoan parasite *Leishmania infantum*. One of the main target organs in CanL is the liver. This complex organ, composed of various highly specialized cell types, has garnered significant attention from the scientific community as a crucial player in innate immune functions. In the context of CanL, liver infection by parasites and the host immune response generated strongly influence the disease outcome. Thus, taking advantage of a co-culture system involving canine hepatocytes and *L. infantum*-infected autologous Kupffer cells (KCs), allowing cell-to-cell interaction, the current report aims to shed light on the hepatocyte-KCs immune interaction. The co-culture of infected KCs with hepatocytes revealed a vital role of these cells in the activation of a local immune response against *L. infantum* parasites. Although KCs alone can be immunologically silenced by *L. infantum* infection, the cell-to-cell interaction with hepatocytes in co-culture can lead to local immune activation. In co-culture it was observed gene expression increased the number of innate immune receptors, specifically cell membrane TLR2 and cytoplasmatic NOD1 along with high TNF-α generation. Altogether, these results suggest that the immune response generated in co-culture could induce the recruitment of other circulating cells to contain and contribute to the resolution of the infection in the liver. This work also enhances our understanding of the liver as a vital organ in innate immunity within the context of CanL.

## 1. Introduction

Leishmaniasis ranks among the most significant vector-borne parasitic diseases distributed globally, alongside malaria and lymphatic filariasis. Estimated to cause 700,000 to 1 million new human infections per year globally and being endemic in 99 countries or territories, leishmaniasis is also considered to be an underreported neglected tropical disease (NTD). Associated with malnutrition and inadequate immune response, as well as marginalized communities, poor housing conditions, and low financial resources, leishmaniasis represents a complex public health problem [1,2]. *L. infantum* is the aetiological agent of human zoonotic visceral leishmaniasis (ZVL) and canine leishmaniasis (CanL), a parasitic disease of major public and veterinary health concern since both ZVL and CanL are life-threatening conditions that may be fatal if not medically treated [3,4]. Dogs play a crucial role in the transmission and maintenance of the parasite. They constitute the main peridomestic reservoir of the parasite to human populations and by exhibiting intense cutaneous parasitism, dogs can facilitate the parasite transmission to the sandfly vector. Consequently, the presence of *L. infantum*-infected dogs in urban environments leads to increased interactions between the vector and parasitized dogs, resulting in a rise in VL cases [2,5]. Additionally, climate change is likely to increase the risk of leishmaniasis in the Mediterranean region, posing several health challenges [6,7].

As in most infections, the complex interaction of the parasite with the host immune system determines the infection outcome. Traditionally, *Leishmania* sp. is described as an intracellular parasite, being macrophages its primary target cells. Once inside macrophages, parasites can manipulate the cell metabolism and immunological status in order to favor its own survival and dispersion in the host, by avoiding the activation of potential leishmanicidal mechanisms. Starting by invading and subverting the skin’s immune response, *L. infantum* parasites migrate in the host to inner organs such as the spleen and liver [8]. In recent years, this complex organ, which is constituted by several different types of highly specialized cells, has attracted the scientific community’s attention as a key player in innate immune functions. In the context of CanL, host immune response has been described as organ-specific and the ability of the canine liver to control and restrain parasite dissemination has been linked to parasite control and asymptomatic infection [9]. Thus, a deeper understanding of the mechanisms the liver employs to control and restrain parasite dissemination can contribute to developing more effective therapeutic strategies, improving disease management, and enhancing our overall knowledge of host–parasite interactions.

Hepatocytes constitute the most abundant liver cell type, make up over 80% of liver cells, and are the major factor responsible for the life-supporting metabolic functions of this organ. Hepatocytes can perform several key immune functions, such as the secretion of acute-phase proteins into the bloodstream. During the acute phase or systemic inflammatory response, hepatocytes are also able to generate and respond to various pro-inflammatory cytokines, including interleukin (IL)-6, IL-1β, IL-22, tumor necrosis factor (TNF)-α, and interferon (IFN)-γ, which can stimulate hepatocytes to produce high levels of complement and acute phase proteins, as well as favor the expression of pattern recognition receptors (PRRs) [10,11,12]. Interestingly, the liver is home to the larger tissue-macrophage community in the mammal body, the Kupffer cells (KCs) [13]. These cells are, in turn, not only the target cells for *L. infantum* infection and replication but are also the cells responsible for the immune surveillance of the organ. KCs are primarily located in the hepatic sinusoids, where their strategic position allows them to efficiently conduct immune surveillance on pathogens. These cells express several innate immune receptors and, upon activation, can synthesize a myriad of cytokines and chemokines. However, KCs exhibit a predominantly immuno-tolerant phenotype shaped by the liver’s naturally tolerogenic microenvironment [14]. This tolerance is crucial to prevent inappropriate immune responses to immunoreactive substances entering the liver from the bloodstream. Canine liver-specific immune response during *Leishmania* infection has been previously addressed by our group [15,16,17,18], highlighting both the immunological role of the liver and its epidemiological significance. The authors have reported that KCs are targeted by *Leishmania* parasites, which induce an anergic state in these immune cells [15,18]. Moreover, hepatocytes appear to play a crucial role in orchestrating the liver’s immune response as these cells can sense the parasite, induce the PRRs gene expression, specifically the cytoplasmatic nucleotide oligomerization domain (NOD)1 and NOD2 and the cell membrane Toll-like receptor (TLR)2, and generate a mix of pro- and anti-inflammatory cytokines, initiating an anti-*Leishmania* immune response [16,17].

Thus, the present study explores the close immune relationship between the two major liver cell populations in the context of *L. infantum* infection, taking advantage of a co-culture system to enable a more detailed analysis of cell-to-cell immune interaction. The results demonstrate that the interaction between hepatocytes and *L. infantum*-infected KCs constitutes key events, favoring the regulation of the local immune response and limiting parasite spread in the liver. Overall, these insights could also pave the way for new approaches to preventing and treating leishmaniasis by targeting specific immune pathways within the liver.

## 2. Materials and Methods

### 2.1. Isolation of Kupffer Cells and Hepatocytes

Canine KCs and hepatocytes were isolated from the same dog using two different liver lobules. The use of autologous cells decreases the risk of immune cross-reaction and ensures cell-to-cell recognition. All dogs used in the study were clinically healthy and tested negative for canine vector-borne diseases, such as *Leishmania infantum* (by indirect immunofluorescence and real-time PCR), *Babesia/Theleria* spp. *Ehrlichia/Anaplasma* spp. (by real-time PCR), as well as for *Dirofilaria immitis*, *Mycoplasma haemocanis*, and *Rickettsia* spp. (detection in blood). Also, hematological (hemogram) and biochemical tests for evaluating liver function, such as total bilirubin quantification, aspartate aminotransferase, and alanine aminotransferase levels, as well as alkaline phosphatase and bile acid, were assessed in the bloodstream. All animals evidenced normal values for hematological and biochemical parameters. KC isolation and culture are described in detail by Rodrigues and colleagues [15]. In summary, a liver lobule was sliced into Gey’s balanced salt solution (Sigma Aldrich, St. Louis, MO, USA) supplemented with 0.2% (*m*/*v*) pronase (Sigma-Aldrich) and 0.8 µg.mL^−1^ DNAse (Roche, Basel, Switzerland) and left to incubate. After washing, the single-cell suspension was overlaid into a double Percoll^®^ (GE Healthcare Bio-Sciences AB, Uppsala, Sweden) gradient and centrifuged. KCs were collected from the intermediate fraction and were washed and resuspended in RPMI medium (BioWhittaker Lonza, Basel, Switzerland) supplemented with 10% heat-inactivated fetal bovine serum (FBS, Sigma-Aldrich), L-Glutamine 0.2 mM (Merck, KGa, Darmstadt, Germany), 100 U.mL^−1^ of penicillin and 100 µg.mL^−1^ of streptomycin (Sigma-Aldrich). The cells obtained were viable and were incubated for 7 days in a six-well plate (VWR) with supplemented RPMI and macrophage colony-stimulating factor (10% *v*/*v* M-CSF). Canine hepatocytes were isolated as described by Rodrigues and colleagues [16,17]. Briefly, collagenase H (Roche Diagnostics, Mannheim, Germany) was used for tissue breakdown, and recovered cells were resuspended in Williams’ E medium (Sigma-Aldrich, Saint Louis, MO, USA) and then overlaid into a solution of 25% Percoll^®^ (GE Healthcare Bio-Sciences AB, Uppsala, Sweden) and centrifuged. After confirming the viability of recovered hepatocytes, these cells were cultured in supplement Williams’ E medium [Penicillin/streptomycin, 1.4 µM hydrocortisone (Sigma-Aldrich), 15 mM HEPES (Sigma-Aldrich), 1 mM sodium pyruvate (Sigma-Aldrich), 1 mM non-essential amino acids (NEAA) (Biochrom GmbH, Irvine, UK), 40 µg.mL^−1^ gentamycin (Sigma-Aldrich), and 10% FBS].

### 2.2. Parasites and Kupffer Cells Infection

*L. infantum* (MHOM/PT/89/IMT151) promastigotes were kept in Schneider medium with L-glutamine, 10% FBS, and penicillin-streptomycin (SCHN, Sigma-Aldrich). Only virulent parasites with fewer than five passages in culture were used [19]. *L. infantum* axenic amastigotes were differentiated as described by Rodrigues and colleagues [15], using stationary phase virulent promastigotes inoculated into pH 5.5 complete SCHN medium and supplemented with 2% of filtered human urine. Amastigote differentiation was confirmed morphologically by scanning electronic microscopy (JEOL5200-LV, JEOL, Ltd., Akishima, Tokyo, Japan). For amastigote infection, KCs concentration was estimated, and cells were exposed to *L. infantum* amastigotes at a parasite-to-cell ratio of 3:1 and left to incubate for 1.5 h, 3 h, and 5 h. Cells were observed under an inverted microscope (Olympus, CKX41, Waltham, MA, USA), and photographs were taken with an Olympus CS30 camera.

### 2.3. Flow Cytometry

To observe the dynamic of *L. infantum*-axenic amastigote infection of KCs, samples from seven dogs (*n* = 7) were used. After 7 days of incubation, cells were detached from the plate, counted, and exposed to *L. infantum* GFP-axenic amastigotes at a parasite-to-cell ratio of 3:1. Cells were incubated for 1.5 h, 3 h, and 5 h. In parallel, non-infected KCs were stained with a polyclonal mouse anti-human monocyte/macrophages FITC antibody (AbD Serotec clone MAC387) that recognizes an intracellular cytoplasmatic protein L1 or calprotectin, that is present in granulocytes, monocytes, and tissue macrophages, allowing the identification of KC population and gating. Non-infected and non-stained cells and parasites were also used as assay controls. Cell acquisition was performed on a FACS Calibur cell analyzer (BD Biosciences, Heidelberg, Germany). At each time point, at least duplicate cell samples were collected and analyzed.

### 2.4. Scan Electron Microscopy (SEM)

To confirm amastigote differentiation in axenic conditions, parasites were prepared for SEM by fixation [2% paraformaldehyde (*m*/*v*) in 1× PBS] as described by Rodrigues and colleagues [16]. Additionally, amastigotes were dehydrated in a graded ethanol series [30, 50, 70, 80, 90, and 100% (*v*/*v*)], dried using the critical point drying method, coated with gold-palladium, and mounted on stubs. Preparations were observed under a scanning electronic microscope (JEOL5200-LV), and digital images were acquired.

### 2.5. Co-Culture System

Co-cultures of *L. infantum*-infected KCs and autologous hepatocytes were established using five dog samples (*n* = 5). After amastigote infection, KCs washed with 1× PBS were detached from the plate by thermal shock and gentle scrape. Cells were added to hepatocytes, generating a co-culture. Cell viability and concentration were assessed in Neubauer’s chamber using trypan blue staining. Co-cultures were established with autologous cells and incubated for 1.5 h, 3 h, 5 h, and 24 h in a cell incubator at 37 °C in a humidified atmosphere with 5% CO_2_. In parallel, (i) non-infected KCs (*n* = 5) and (ii) KCs infected with amastigotes (*n* = 6), as well as (iii) non-stimulated hepatocytes (*n* = 10) were prepared in order to allow comparations with the established co-cultures. Cells status were followed using an Olympus CKX41 inverted microscope and images were captured with an Olympus CS30 camera. At each time point, samples from the different experimental settings were collected in duplicate (minimum) and analyzed.

### 2.6. Real-Time PCR

Gene expression of PRRs (NOD1, NOD2, TLR2, TLR4, and TLR9) and cytokines [IL-10, IL-4, IL-12p40, transforming growth factor (TGF)-β and TNF-α] were quantified by real-time polymerase chain reaction quantitative method (RT-qPCR). The protocols and primers used were described by Rodrigues and co-workers [15,16]. In summary, RNA extraction was made using NZY Total RNA Isolation Kit (Nzytech genes and enzymes, Lisbon, Portugal), and cDNA synthesis was performed using an NZY First-strand cDNA Synthesis Kit (Nzytech genes and enzymes) following the manufacturer’s protocol. RT-qPCR was performed in a 7500 FAST Real-Time PCR System thermal cycler (Applied Biosystems, Foster City, CA, USA). Amplification was carried out in a total volume of 20 µL, containing 2 µL of cDNA, 10 µL of SensiFAST SYBR Lo-ROX (Bioline Reagents Ltd., Meridian Bioscience, London, UK), and primers (20 pmol.µL^−1^). External cDNA standards were produced for all analyzed genes by cloning PCR fragments into a pGEM^®^-TEasy Vector (Promega, Madison, WI, USA) as described by Rodrigues et al. [20]. Each sample, standard, and non-template were analyzed in duplicate. The number of copies of each gene and sample was normalized to the housekeeping gene β-actin to standardize differences in the relative quantities of initial cDNA in the sample.

### 2.7. Statistical Analysis

The non-parametric Wilcoxon test for two related samples was used to assess the variations between time points (1.5 h, 3 h, 5 h, and 24 h) and the following experimental conditions: (i) non-infected KCs (*n* = 5), (ii) KCs infected with *L. infantum* amastigotes (*n* = 6), (iii) non-stimulated hepatocytes (*n* = 10) and (iv) *L. infantum* infected KCs co-cultured with autologous hepatocytes. Data analysis was performed using the software GraphPad Prism 9 (GraphPad Software, Boston, MA, USA) and considering a significance level of 5% (*p* < 0.05). This study revealed a diverse dispersion of values with several outliers, which reflect the natural diversity within the canine population, as no restrictions on breed, gender, or age were applied.

### 2.8. Ethical Considerations

The present work was conducted using samples from deceased animals. Liver samples were obtained from a local shelter after dogs were euthanized due to aggressiveness and lack of adoption. Since no live animals were used, there was no need to consult the Commission on Ethics and Animal Wellbeing for advice for this study.

## 3. Results and Discussion

### 3.1. Co-Cultures Do Not Extensively Activate PRRs

**Figure 1:**
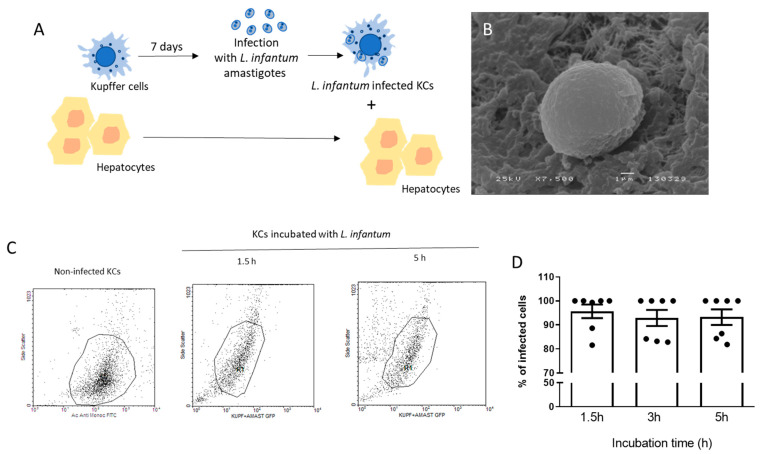
Experimental design and Kupffer cells infection with *L. infantum* amastigotes. (**A**) After isolation, canine KCs were cultured for 7 days to achieve maximum proliferation and differentiation and exposed for 5 h to axenic *L. infantum* amastigotes. After infection, cells were added to hepatocytes, establishing a co-culture. (**B**) Scan electron microscopy of axenic *L. infantum* amastigotes. (**C**) flow cytometry analysis of infected KCs. Non-infected KCs were stained with anti-human monocyte/macrophage FITC antibody. R1 corresponds to the KCs population infected with GFP-amastigotes. (**D**) percentage of infected KCs during different incubation times. Individual values are represented by dots and bars representing mean and standard error (*n* = 7).

**Figure 2:**
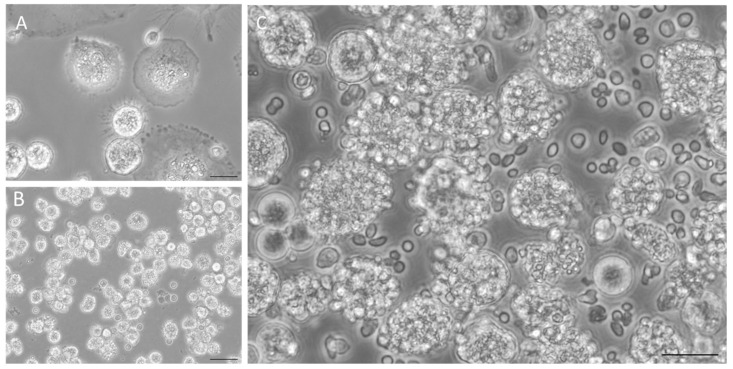
Hepatocytes-KCs co-cultures. (**A**) Canine KCs after 7 days in culture (400× magnification) and (**B**) autologous hepatocytes (200× magnification). (**C**) Co-culture of *L. infantum*-infected KCs with autologous hepatocytes (400× magnification). Scale bar 20 µm.

To simulate the natural infection in the liver and assess if the presence of hepatocytes might boost local anti-leishmania immune activation signals, co-cultures of *L. infantum*-infected KCs and autologous hepatocytes were performed (Figure 1A). As KCs are the natural amastigote-target cells, *L. infantum* amastigotes were in vitro differentiated and used to infect KCs, thus simulating the natural infection of the organ. The morphological differentiation from motile promastigotes to round-shape amastigotes, which occurs naturally during the mammal infection, was triggered in vitro by decreasing the pH and increasing temperature, representing both the intracellular parasitophorous vacuole and mammal body temperature. Altogether, these conditions induce the parasite to change into its intracellular form, characterized by a rounded shape and loss of the free flagellum, as observed by SEM photography (Figure 1B). Axenic amastigotes were able to infect KCs (Figure 1C,D) after 1.5 h of exposure and sustained the infection up to 5 h of incubation. However, at 24 h of exposure to the parasite, high levels of cell lysed KCs were observed [15]; thus, exposure to axenic amastigotes was limited to 5 h maximum time. Hence, isolated KCs (Figure 2A) were exposed to axenic amastigotes during 5 h to ensure KCs infection and then were added to autologous hepatocytes (Figure 2B), generating a co-culture (Figure 2C). Altogether, this experimental design replicated natural liver infection and enabled the continuation of the study.

**Figure 3:**
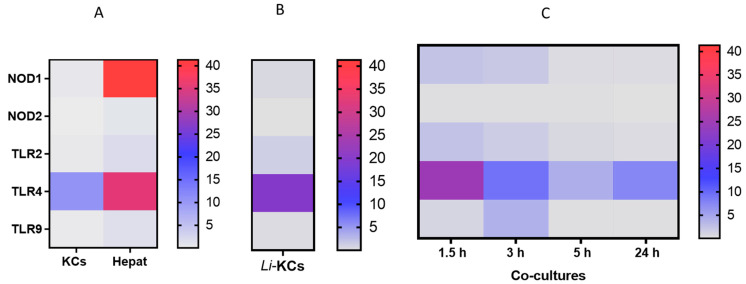
Gene expression of innate immune receptors analyzed by real-time PCR. (**A**) Resting KCs (*n* = 5) and hepatocytes (Hepat) (negative control) (*n* = 10); (**B**) KCs infected with *L. infantum* amastigotes (Li-KCs) for 5 h (*n* = 6); (**C**) co-culture of hepatocytes and KCs infected with *L. infantum* amastigotes for 5 h incubated for 1.5 h, 3 h, 5 h and 24 h (*n* = 5). Values represent the median copy number/1000 copies of β-actin.

Then, PRRs gene expression was analyzed in resting KCs and hepatocytes (negative control), in *L. infantum*-infected KCs, and hepatocytes and *L. infantum*-infected KCs that were in co-culture. NODs and TLRs constitute some of the most important classes of PRRs in the context of leishmaniasis. In the current study, resting KCs and hepatocytes express different PRRs. KCs appear to present high TLR4 gene expression, and hepatocytes naturally exhibited high levels of NOD1 and TLR4 mRNA, and, to a lesser extent, TLR2, TLR9, and NOD2 mRNA accumulation (Figure 3A). Interestingly, it was described that primary hepatocytes constitutively express RNA for all TLRs but tightly regulate the presence of TLR proteins to prevent excess immune activation [21,22].

The amastigote infection of KCs over a 5 h period does not induce a distinct pattern of PRR gene expression compared to resting KCs (Figure 3B). Thus, it is noteworthy to observe the strong immune repression exerted by the parasite on infected KCs, as the gene expression of the assessed PRRs on *L. infantum*-infected KC is similar to or lower than resting KCs [15]. Interestingly, in co-cultures, it was observed that several of the accessed PRRs registered an early, transient increase (1.5 h) in their gene expression when compared to non-infected KCs or *L. infantum*-infected KCs alone (P_TLR2 1.5 h_ = 0.0020) (Figure 3C). However, after 5 h of co-culture, the PRR gene expression registered a downregulation compared to the early time point (P_TLR4 1.5 h–5 h_ = 0.0371 and P_TLR9 1.5 h–5 h_ = 0.0420).

Altogether, these results might suggest that infected KCs exert immune regulation on surrounding hepatocytes, and avoid excessive activation, raising the hypothesis that parasites use KCs to extend their immune suppression to hepatocytes.

### 3.2. Co-Cultures Can Generate Key Cytokines and Orchestrate an Immune Response

**Figure 4:**
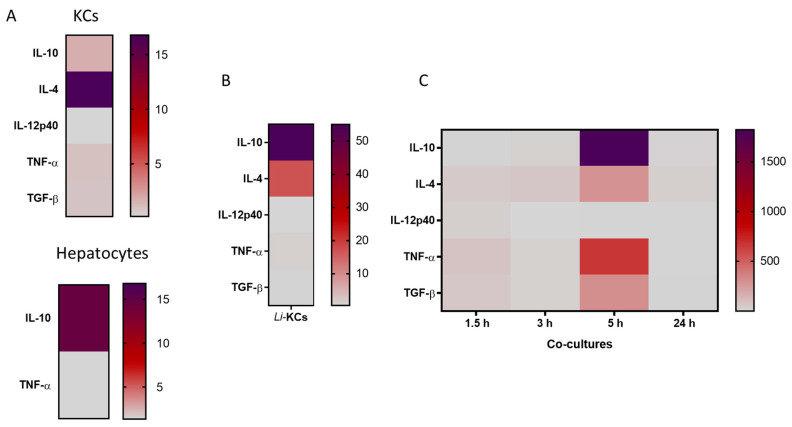
Gene expression of pro-inflammatory and anti-inflammatory key cytokines by real-time PCR. (**A**) Resting KCs (*n* = 5) and hepatocytes (negative control) (*n* = 10); (**B**) KCs infected with *L. infantum* amastigotes (Li-KCS) for 5 h (*n* = 6); (**C**) co-cultures of hepatocytes and KCs infected with *L. infantum* amastigotes for 5 h incubated for 1.5 h, 3 h, 5 h and 24 h (*n* = 5). Values represent the median copy number/1000 copies of β-actin.

The immune response generated was evaluated by analyzing the gene expression of a panel of cytokines that play key roles in initiating and coordinating the immune response. Both resting KCs and hepatocytes exhibit basal gene expression of cytokines, mainly IL-4 and IL-10, respectively (Figure 4A). These cytokines have important anti-inflammatory functions and may be associated with the immunotolerant microenvironment of the liver. *L. infantum* amastigotes induced KCs to upregulate IL-10 and IL-4 gene expression, reflecting a potential immune suppression promoted by the presence of the intracellular parasite (Figure 4B). Usually, non-resolving infections are associated with the production of anti-inflammatory cytokines such as IL-4, IL-13, and/or IL-10. This immune microenvironment directs the polarization of infected macrophages towards the M2 activation state, leading to a permissive immune response that may also promote tissue repair [23].

Co-cultures can generate high levels of cytokines involved in the orchestration of an immune response. Pro-inflammatory TNF-α is a key cytokine in the liver immune environment strongly involved in liver inflammation. In co-cultures, a consistent upregulation of TNF-α was transiently observed after 5 h of incubation (Figure 4C), which was higher than resting cells (KC or hepatocytes) and infected KCs (P_5 h_ = 0.0042), suggesting an effective inflammatory activation resulting from the interplay between hepatocytes and infected KCs. IL-10 was also upregulated in co-cultures over time (P_5 h_ = 0.0078) (Figure 4C). Similarly, the levels of IL-10 gene expression were higher when compared with resting cells (resting KCs or hepatocytes) and infected KCs (P_5 h_ = 0.0351). Other important anti-inflammatory cytokines, such as IL-4 and TGF-β, were also upregulated in co-cultures from 1.5 h to 5 h. Altogether, these results suggest the launch of an active and well-balanced immune response against *L. infantum* parasites.

### 3.3. The Interaction of L. infantum-Infected KCs with Hepatocytes Is Key in Regulating Local Immune Response

**Figure 5:**
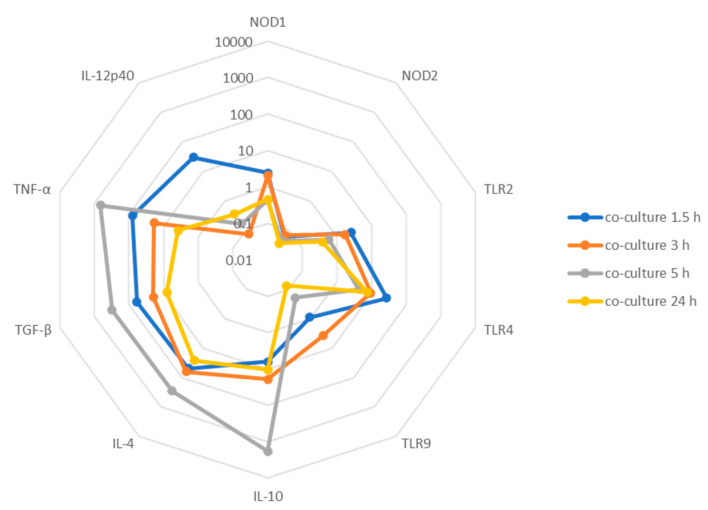
Interplay of canine *L. infantum*-infected KCs and hepatocytes (co-culture) over 24 h. Values represented as log of median copy number/1000 copies of B-actin (*n* = 5).

Apart from the role of performing life-supporting metabolic functions, the liver, in particular the hepatocytes, play a key role in controlling systemic innate immunity via the release of PRRs, such as collectins, pentraxins, ficolins, lipid transferases, peptidoglycan recognition proteins, and the leucine-rich repeat receptor, as well as complement components found in plasma [24]. During an acute phase or systemic inflammatory response, various pro-inflammatory cytokines, including IL-6, IL-1, TNF-α, and IFN-γ, can activate hepatocytes to produce high levels of complement proteins, secrete PRRs, and become immune-activated and predisposed to inflammation. Hepatocytes not only are the major source of secreted PRRs but also express membrane-bound PRRs, such as TLRs or cytoplasmatic NODs, similarly to what is observed in other immune cells, such as the KCs. Indeed, the analysis of the progression of co-cultures over time (Figure 5) results in the generation of NOD1, TLR2, and TLR4 at an early time point (1.5 h), suggesting the engagement of these PRRs in the CanL.

**Figure 6:**
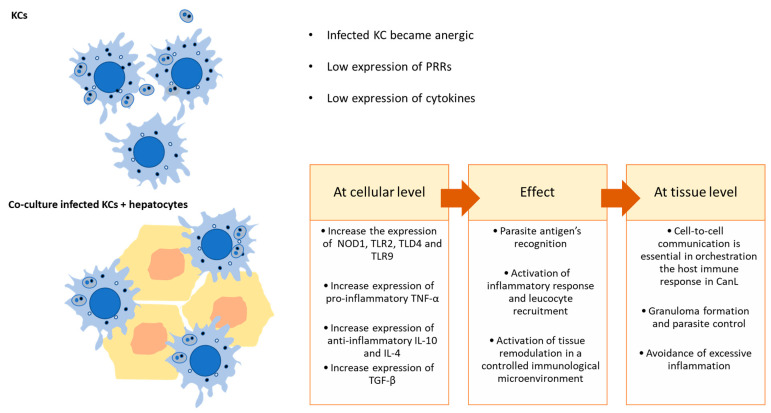
Proposed model for KCs interaction with hepatocytes in the context of CanL.

Hepatocytes were found to express messenger RNAs for all described TLRs, but their functions on hepatocytes are still being determined, as well as the role of PRRs in host defenses against CanL [25,26]. The co-culture of infected KCs with hepatocytes highlights the vital role of these cells in activating the liver’s immune response against *L. infantum* parasites (Figure 6). Kupffer cells constitute the parasite target cells, but when facing *L. infantum* parasites, these cells exert low immune action and can fall into a parasite-induced anergic state [18]. However, the surrounding liver cells, as is the case of hepatocytes, can sense the threat and activate local immune response, as observed by the early generation of pro-inflammatory TNF-α gene expression (Figure 5). Such gene expression of pro-inflammatory cytokines can initiate the recruitment of circulating immune cells, such as lymphocytes from the bloodstream. The recruitment of these cells in a microenvironment of immune activation leads to an effective control of the parasite in the organ (Figure 6).

Although the liver is a target organ in ZVL and CanL, the infection in the liver is normally self-containing in asymptomatic individuals in contrast to the spleen, which stays chronically infected, allowing parasite replication [9,27]. This control is achieved by the formation of effective granulomas, isolating parasites, and displaying an active pro-inflammatory immune response with effector T cells. This cellular microenvironment is characterized by high IFN-β, IL-12, and TNF-α production. On the other hand, in symptomatic animals, liver granulomas are found to be non-organized and to have heavily parasitized cells in an ineffective immune microenvironment [28]. The generation of high levels of TNF-α in co-culture is therefore a strong indication of the activation of an immune response similar to in vivo activation that leads to granuloma formation. TNF-α is a pleiotropic cytokine associated with liver pathogenesis, constituting cell survival signaling, inducing hepatocyte proliferation, and also regulating metabolic processes [29]. In the liver, this cytokine has been described as having a dual role, either aggravating or alleviating injury, being crucial to an effective inflammatory immune response, by activating NF-κB, thus being fundamental in anti-Leishmania actions [30]. A protective immune response in ZVL is strongly dependent on TNF-α generation, as this cytokine is required for the expansion of IFN-γ^+^ CD4^+^ T cells, with several studies suggesting that TNF-α is essential to disease control [31]. While inflammation is crucial for immune-mediated defense against most pathogens, regulatory mechanisms are needed to prevent excessive damage to host tissues. The liver is the major metabolic organ, and an extensive inflammatory response may have serious consequences for the host. So, hepatocytes, while transiently immune activated, as shown by the increase in IL-12 and TNF-α, rapidly induce immune tolerance to avoid major damage to the organ and the host, as seen by the increase in IL-10, IL-4, and TGF-β generation (Figure 5). IL-10 is a pleiotropic cytokine with a strong immunoregulatory role, essential to balance an inflammatory response to prevent extensive tissue damage to the organs [32]. IL-10 expression in the liver is also associated with the transition of acute inflammation to chronic conditions [33]. This chronicity is common in ZVL and CanL. Interestingly, IL-10 has gained significant interest due to its potential therapeutic role in liver fibrosis. Liver fibrosis is a consequence of an unbalanced hepatic immune response and compromises liver functions. IL-10 is described to have an anti-fibrotic effect that improves NK cell immune function, including activation, cytotoxicity, development, and migration [34]. Thus, our co-culture model might be useful for future studies to provide valuable insights into the mechanisms through which IL-10 regulates NK cells to limit the progression of liver fibrosis. TGF-β plays pleiotropic functions in the control of proliferation, differentiation, and immunosuppression of cells in different tissues [35]. In leishmaniasis, the TGF-β1 is the most studied and is associated with the enhancement of polyamine production, favoring parasite survival [31]. IL-4 is another vital anti-inflammatory (Th2) cytokine that appears to be key in ZVL and CanL. It functions as a growth factor and is crucial for activating mast cells and naive CD4^+^ Th2 lymphocytes, which can produce other anti-inflammatory cytokines, such as IL-5, IL-10, and IL-13. It also triggers macrophages to undergo alternative (M2) activation, with low production of reactive oxygen species and nitric oxide, resulting in parasite survival and persistence of infection [31,36,37].

## 4. Conclusions

Inferring from hepatocytes *L. infantum*-infected KCs co-cultures, these cells can sense the parasite infection and become activated, producing a burst of pro-inflammatory cytokines. However, the tendency of liver cells to avoid inflammation rapidly leads to the generation of anti-inflammatory immune signals to induce immunological permissiveness/tolerance. Nevertheless, this initial inflammatory burst might be enough to call neutrophils, lymphocytes, and macrophages from the bloodstream into the liver to resolve the infection and, in the particular case of *L. infantum* infection, create the resistance-associated liver structure: the granuloma. Although the co-culture system allows one to infer close cell-to-cell communication and immune signaling in a controlled and stable microenvironment, it does not represent the entire complexity of the liver or the organism in cell behavior due to artificial conditions, such as lack of blood flow, diminished cell diversity, and impaired cell ratio, or absence of extracellular matrix components, which are not fully replicated in co-cultures. Also, CanL is a multiorgan complex disease; thus, it can be a challenge to mimic the overall pathology. Co-cultured cell systems often have a limited lifespan and may not maintain their physiological characteristics over extended periods. This limits the ability to study long-term interactions and chronic processes. In particular, primary cell co-cultures rapidly enter in the senesce phase, and ethical sourcing of primary cells (from animals or humans) can restrict their availability. Nevertheless, it constitutes an effective tool contributing to understanding how liver cells react and orchestrate the immune response against *L. infantum* parasites. Furthermore, co-culture systems remain a valuable methodology for understanding cell–cell interactions by studying specific aspects of cellular behavior. These in vitro cell systems can be conjugated with other experimental approaches to detail the cellular immune responses.

Our findings confirm that hepatocytes are immune active cells, being key players in the liver’s early immune response to *L. infantum* infection and contributing to parasite control. Altogether, these findings add new knowledge for a better understanding of the liver as an innate immunological key organ, as well as bringing new awareness on host–parasite immune interaction. Also, since the liver’s immune functions appear to be conserved across mammals, the research in dogs can offer comparative insights relevant to other species, including humans, closing the gap between veterinary and human medicine in the context of CanL e ZVL, respectively. Thus, the present study may contribute to the development of innovative immune-based therapies, targeting parasite clearance and modulating the liver’s immune response, paving the way for more personalized veterinary care, considering the specific immune profile of individual dogs, and improving disease outcomes.

## Data Availability

The raw data supporting the conclusions of this article will be made available by the authors on request.
